# Induction of Chirality in Atomically Thin ZnSe and CdSe Nanoplatelets: Strengthening of Circular Dichroism via Different Coordination of Cysteine-Based Ligands on an Ultimate Thin Semiconductor Core

**DOI:** 10.3390/ma16031073

**Published:** 2023-01-26

**Authors:** Daria A. Kurtina, Valeria P. Grafova, Irina S. Vasil’eva, Sergey V. Maksimov, Vladimir B. Zaytsev, Roman B. Vasiliev

**Affiliations:** 1 Department of Chemistry, Lomonosov Moscow State University, 119991 Moscow, Russia; 2 A. N. Bach Institute of Biochemistry, Research Center of Biotechnology of the Russian Academy of Sciences, Leninsky Ave. 33, Bld. 2, 119071 Moscow, Russia; 3 Department of Physics, Lomonosov Moscow State University, 119991 Moscow, Russia; 4 Department of Materials Science, Lomonosov Moscow State University, 119991 Moscow, Russia

**Keywords:** 2D semiconductors, chirality, CdSe nanoplatelets, ZnSe nanoplatelets, circular dichroism, excitons, ligand exchange

## Abstract

Chiral nanostructures exhibiting different absorption of right- and left-handed circularly polarized light are of rapidly growing interest due to their potential applications in various fields. Here, we have studied the induction of chirality in atomically thin (0.6–1.2 nm thick) ZnSe and CdSe nanoplatelets grown by a colloidal method and coated with L-cysteine and N-acetyl-L-cysteine ligands. We conducted an analysis of the optical and chiroptical properties of atomically thin ZnSe and CdSe nanoplatelets, which was supplemented by a detailed analysis of the composition and coordination of ligands. Different signs of circular dichroism were shown for L-cysteine and N-acetyl-L-cysteine ligands, confirmed by different coordination of these ligands on the basal planes of nanoplatelets. A maximum value of the dissymmetry factor of (2–3) × 10^−3^ was found for N-acetyl-L-cysteine ligand in the case of the thinnest nanoplatelets.

## 1. Introduction

Chirality is a unique phenomenon of nature in which a structure/molecule is non-superposable with its mirror image. In addition to its important role in biochemical and chemical processes, chirality leads to different interactions of molecules and structures with right- and left-handed circularly polarized light. Recently, chiral colloidal nanoparticles and nanostructures [1] have been of great interest, demonstrating different absorption of right- and left-handed circularly polarized light (circular dichroism, CD) or rotation of the light polarization plane (optical activity), as well as the emission of photons of a given circular polarization (circularly polarized luminescence). Chiral nanostructures have promising applications in biochemical, photonic, and optoelectronic technologies, such as enantioselective separation and asymmetric catalysis, display technologies, and spintronics (for example, the chiral-induced spin selectivity effect) [2,3,4,5,6].

Chiral optical properties, including optical activity and circular dichroism, are possessed by nanoparticles having an intrinsic chirality of the material, for example, mercury sulfide [7]. To obtain chiral nanoparticles from achiral semiconductors, chirality induction is used, i.e., the creation of optical activity of nanoparticles induced by chiral ligands, which was first shown for CdS nanoparticles coated with D- or L-penicillamine [8]. Later, optical activity induced by ligands was shown in a number of works for CdSe and CdTe nanoparticles coated with different types of chiral ligands [9,10,11,12,13]. An important role in the chiroptical properties of nanoparticles and nanostructures is played by their size and shape. For example, CdSe nanoparticles coated with chiral penicillamine ligand exhibit an optical activity that depends on the size of the nanoparticle, with the magnitude of the optical activity (related to dissymmetry g-factor) decreasing with increasing size due to a decrease in the surface to volume ratio [14]. This indicates the possibility of an increase in the dissymmetry g-factor for anisotropic nanostructures, as was found for CdSe nanorods with L/D-cysteine ligands, for which the g-factor increased with increasing rod anisotropy [15]. A further increase in the optical activity is possible for two-dimensional nanostructures; one representative of which—colloidal semiconductor nanoplatelets (NPLs)—confined excitons quantum-mechanically in one dimension. As shown in [16], a high dissymmetry factor of up to 8 × 10^−4^ was achieved for CdSe NPLs with a thickness of 3–5 monolayers (ML, pairs of atomic planes) coated with L/D-cysteine ligands, which exceeds that commonly reported for spherical nanoparticles (g-factor about 10^−5^). Later in our work [17], we showed that a record dissymmetry factor g of 3 × 10^−3^ among semiconductor A^II^B^VI^ nanoparticles can be achieved in atomically thin CdSe NPLs having 2 ML thickness and coated with N-acetyl-L/D-cysteine. At the same time, the value of the g-factor was influenced by a number of factors, such as the type of chiral ligands, the density of chiral ligands, and the coordination and binding of chiral ligands [18]. For example, it was shown for CdSe nanoplatelets capped with L/D-cysteine that the crystal structures of wurtzite and zinc blend demonstrate induction of chirality with different signs [19]. The hexagonal crystal structure showed a 10-times-greater CD signal compared with those with a cubic structure, which was explained by their different dipole moment and polarization. Also, in addition to having a thickness of only a few atoms and a 2D electronic structure of NPLs, flat basal planes with a slightly different lattice parameter compared to the ligand footprint led to the strong tensile strain of the semiconductor [17], providing the densest packing of ligands, which is not observed for nanoparticles with a different morphology. This should change the interaction between the ligands and the semiconductor and affect the coordination of the ligands. Yet, understanding how the interplay of anisotropy, crystal structure, and ligand coordination in 2D nanoplatelets leads to strong optical activity and a high CD signal remains a challenge.

Here, we report a study of the induction of chirality in atomically thin ZnSe and CdSe NPLs upon native long-chain ligands exchanged with chiral L-cysteine (L-Cys) and N-acetyl-L-cysteine (L-AcCys) ligands. We synthesized ZnSe and CdSe NPLs with ultimately thin thicknesses of 0.6−1.2 nm and lateral sizes of 100–200 nm in order to achieve strong quantum confinement and pure 2D regime for excitons and enhance their interaction with ligands on basal planes of NPLs. We compared the most popular chirality-inducing ligand, L-cysteine, with the more sterically bulky cysteine-derivative N-acetyl-L-cysteine, and studied NPLs with different basal surfaces—CdSe NPLs of the zinc blende modification and ZnSe NPLs of the wurtzite modification. We separately analyzed different Cys and AcCys ligands on the same semiconductor core, and the same ligands on the different CdSe and ZnSe cores. The composition and coordination of ligands on the basal planes of the NPLs were analyzed in detail by Fourier-transform infrared spectroscopy (FTIR). The optical and chiroptical properties of chiral atomically thin ZnSe and CdSe NPLs were studied by absorption, luminescence, and circular dichroism (CD) spectroscopy.

## 2. Materials and Methods

Cadmium acetate dihydrate (Cd(CH_3_COO)_2_∙2H_2_O, ≥98%), zinc acetate dihydrate (Zn(CH_3_COO)_2_∙2H_2_O, ≥98%), oleic acid (OA, 90%), oleylamine (OLA, 90%), octylamine (OTA, 95%), selenium powder (Se, 99.99%), trioctylphosphine (TOP, 90%), 1-octadecene (ODE, 90%), N-methylformamide (NMF, 90%), L-cysteine (L-Cys, ≥99%), N-acetyl-L-cysteine (L-AcCys, ≥99%) and solvents were purchased from Sigma-Aldrich. Cadmium acetate dihydrate was further recrystallized from hot aqueous solution with a small addition of acetic acid to prevent hydrolysis.

Colloidal growth of CdSe NPLs with a thickness of 2.5 ML was carried out similarly to [17]. A quantity of 480 μL of oleic acid and 0.26 g of freshly recrystallized cadmium acetate dihydrate were added to 20 mL of octadecene in a flask. Next, the solution was heated to a temperature of 170–190 °C under argon flow, after which it was degassed for some time. At the end of degassing, the mixture was cooled to the injection temperature (120 °C). A quantity of 200 μL of selenium in trioctylphosphine solution (1 M) was injected rapidly, and the mixture was gradually heated to a growth temperature (160 °C) for an hour. The growth of CdSe nanoparticles lasted 3.5 h on average. After the synthesis was completed, 2 mL of oleic acid was added to the resulting solution as a stabilizer, and the resulting mixture was precipitated by centrifugation (6000–7000 rpm), with acetone as an antisolvent. The precipitate was finally redispersed in hexane. As a result, solutions of light-yellow color were obtained. The growth and purification of CdSe NPLs with a thickness of 3.5 ML was carried out similarly, except with a growth temperature of 200 °C and growth time of 30 min.

Colloidal growth of ZnSe NPLs with a thickness of 4 ML was carried out, with certain changes from [20]. For the synthesis of zinc precursor (zinc oleate), a mixture of 138 mg zinc acetate dihydrate with 0.44 mL OA in 3 mL ODE was heated at 180 °C for 90 min under argon flow. For the growth of NPLs, 59 mg elemental selenium was added to a 25 mL two-neck round-bottomed flask, along with 5 mL octylamine and 7 mL oleylamine. The mixture was degassed at 100 °C for 30 min under argon flow. Then, the reaction mixture was heated up to the growth temperature: 180 °C for 4 ML thickness and 110 °C for 2.5 ML thickness. After that, the zinc precursor was injected into the flask. Three hours later, 2 mL OA was added and the flask was cooled naturally to room temperature. Then, 1 mL TOP was used to dissolve unreacted Se. The synthesized ZnSe NPLs were precipitated by centrifugation (3500 rpm) with acetone as an antisolvent. A white (2.5 ML NPLs) or yellow (4 ML NPLs) precipitate was finally redispersed in 4 mL of hexane.

Ligand exchange with L-cysteine (L-cys) and N-acetyl-L-cysteine (L-AcCys) ligands was carried out using the phase-transfer method and organic solvent method, similarly to [17]. Briefly, in the case of CdSe NPLs, 50 mg of N-acetyl-L-cysteine or L-cysteine was pre-dissolved in 1 mL of tetrahydrofuran (THF) under sonication, and the solution was added to 1 mL of the initial nanoparticle solution (200 μL of as-synthesized CdSe NPLs) in hexane. The exchange was carried out for an hour. After that, the reaction mixture was precipitated by centrifugation with acetone as a precipitator. The procedure was repeated until the native ligand disappeared in FTIR. The resulting nanoparticles covered with AcCys or Cys were dissolved in 2 mL of methanol. In the case of ZnSe NPLs, 200 μL of as-synthesized ZnSe NPLs in hexane was diluted in 1 mL hexane and added to 1 mL methanol solution of 50 mg of acetylcysteine under continuous stirring. After 1 h, a hexane layer was discarded and an equal volume of toluene was added. NPLs were separated by centrifugation at 5000 rpm for 10 min and washed several times with toluene. The procedure was repeated until the native ligand disappeared in FTIR, and ZnSe NPLs covered with AcCys were finally dissolved in 2 mL of methanol.

Transmission electron microscopy (TEM) images were recorded on the LEO19 AB OMEGA microscope (Zeiss, Oberkochen, Germany) operated at 100 kV and the JEOL JEM2100 microscope (JEOL, Tokyo, Japan) operated at 200 kV. Fourier-transform infrared spectroscopy (FTIR) spectra were recorded on a Perkin-Elmer Frontier FTIR spectrometer (Perkin Elmer, Waltham, MA, USA) at room temperature in the 400–4000 cm^−1^ wavenumber range. Samples for analysis were prepared by mixing a drop of NPLs solution or reference Cys or AcCys solution in acetone with KBr powder, followed by pressing into tablets after solvent evaporation. Absorption spectroscopy was carried out in the 200–800 nm wavelength range, with scanning speed 60 nm/min on the Varian Carry50 (Agilent Technologies, Santa Clara, CA, USA) spectrophotometer. Photoluminescence spectra were collected with an LS 55 (Perkin-Elmer, Waltham, MA, USA) fluorescence spectrometer. Circular dichroism (CD) spectra were recorded on a Chirascan spectropolarimeter (Applied Photophysics, Leatherhead, UK) in the 300–500 nm wavelength range, with scanning speed 10 nm/min and 1 nm step (integration time 3 s). Optical measurements were carried out at room temperature from colloidal solutions of NPLs in hexane or methanol diluted to an optical density < 1. Quartz cuvettes (Hellma Analytics, Müllheim, Germany) with 1 cm optical path length were used. Dissymmetry g-factor was calculated as *g = ΔA*/*A =* (*A_L_* − *A_R_*)/*A*, where *A_L_* and *A_R_* are the absorbances of circularly polarized left-handed and right-handed light, respectively, and *A* is the absorbance of unpolarized light.

## 3. Results

### 3.1. Atomically Thin Nanoplatelet Growth and Ligand Exchange

**Figure 1:**
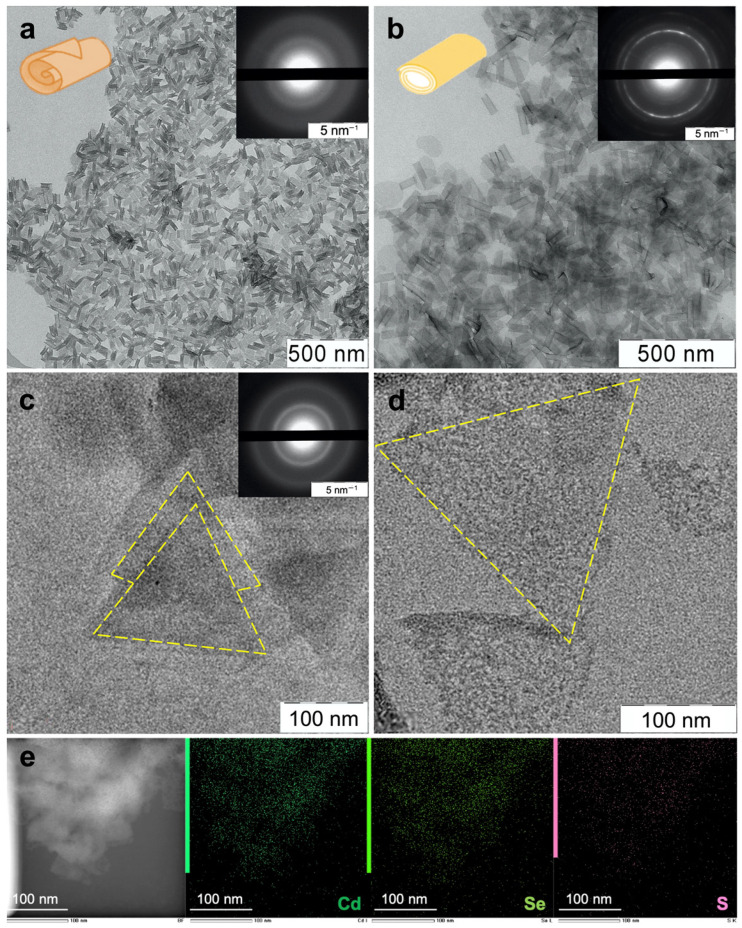
Low-magnification TEM images of (**a**,**c**) as-synthesized scroll-like CdSe394OA and triangular ZnSe293OA NPLs covered with oleic acid ligands and (**b**,**d**) the same NPLs after ligand exchange with L-AcCys. Inserts show electron diffraction images. (**e**) EDX mapping of CdSe394 covered with L-AcCys ligand.

Atomically thin ZnSe and CdSe NPLs were synthesized by the colloidal growth method. To obtain extremely thin CdSe NPLs with zinc blende crystalline modification, we used our previously developed protocol for growth in the ODE–OA–cadmium acetate system at low temperatures [17]. CdSe NPLs with thicknesses of 2.5 and 3.5 ML (0.6 and 0.9 nm) stabilized with an oleic acid ligand were obtained after growth. The lowest excitonic transitions for these samples were at 394 and 463 nm for 2.5 and 3.5 ML thickness, respectively, so we denoted them as CdSe394OA and CdSe463OA. The TEM images of as-synthesized NPLs (Figure 1a and Figure S1a) demonstrate uniformlyshaped and -sized sheets rolled into nanoscrolls due to the effect of spontaneous folding [21,22]. The average lateral size of the NPLs was about 100 nm, similarly to the data of [17]. The modification of zinc blende was confirmed by electron diffraction (ED), which showed a sequence of reflections of cubic CdSe (Figure 1a and Figure S1a, inserts), which is consistent with the results of [17].

At the same time, the growth of ZnSe NPLs under similar conditions turned out to be impossible due to the lower formation energy of zinc chalcogenides compared with cadmium chalcogenides. As was shown [23], the growth of zinc chalcogenide nanoparticles requires the presence of amines. Thus, we conducted the synthesis of ZnSe NPLs in a medium of long-chain amines, similarly to synthesis of the previously studied CdSe nanoribbons of wurtzite modification, also grown in the presence of amines [24]. A similar approach was used in [20] for the synthesis of ZnSe nanoplatelets of the wurtzite modification. Using the system ODE–OLA–OTA–zinc oleate and elemental Se solution in an OTA/OLA mixture as a selenium source, we grew ZnSe NPLs at temperatures of 180 °C. A TEM image (Figure S2a) demonstrates uniform rectangular nanoplatelets 40 × 10 nm in size, similar to [20]. Their thickness can be reported as 4 ML (1.2 nm) by comparison with the data of [20]. To further reduce the thickness of the ZnSe NPLs, we carefully modified the synthesis conditions, reducing growth temperature and growth solution composition. We achieved the growth of a new, thinner population of ZnSe NPLs, not reported in the literature to the best of our knowledge, at a lower growth temperature of 120 °C, in the presence of OA, and changing the ratio of zinc to selenium from 1:2 to 1:1. The TEM image of as-grown NPLs (Figure 1c) demonstrate uniform flat triangular 2D sheets with lateral dimensions of about 200 nm. We estimated the NPL thickness at 0.6 nm, based on nanosheet stack analysis (Figure S2b), and assigned an NPL thickness of 2.5 ML due to the symmetric basal planes that require an additional atomic plane for the wurtzite in the [0001] direction. We denoted 2.5 ML and 4 ML ZnSe samples as ZnSe293OLA and ZnSe347OLA, respectively. We assumed a crystalline modification of wurtzite for the synthesized ZnSe NPLs, which was confirmed by the electron diffraction (Figure S2a, insert) distinctly showing a clear reflection of the wurtzite phase for the 4 ML sample. At the same time, for the 2.5 ML sample, the ED reflections are much weaker due to the extreme thinness, but they indicate a hexagonal modification consistent with the triangular shape of the NPLs, which confirms the wurtzite modification for the 2.5 ML ZnSe samples (Figure 1c, insert).

Ligand exchange of native long-chain OA, OTA and OLA ligands of as-synthesized NPLs with chiral L-Cys and L-AcCys ligands was conducted using the phase-transfer method and organic solvent method. In the case of CdSe NPLs, the organic solvent method followed our earlier-reported protocol in tetrahydrofuran solvent [17]. However, this approach was ineffective in the case of ZnSe NPLs because of the low exchange rate. We developed a phase-transfer method with nonpolar phase (hexane) for as-synthesized NPLs with long-chain ligands and polar phase (methanol) with polar L-Cys and L-AcCys ligands. Ligand-exchanged NPLs transferred to polar phase upon coating with L-Cys and L-AcCys ligands. Unfortunately, L-Cys-covered NPLs were insoluble in any kind of solvent, so we further studied only L-AcCys ligand for ZnSe NPLs. The completeness of the ligand exchange for CdSe and ZnSe NPLs was confirmed by FTIR spectroscopy by the complete disappearance of vibrations from the long-chain OA, OTA and OLA ligands and by significant changes in the FTIR spectra (Figure S3).

The TEM images of ligand-exchanged NPLs (Figure 1b,d) showed the preservation of the 2D morphology and crystal structure. Rolled nanosheets with lateral sizes of about 100 nm were found for CdSe NPLs coated with the L-AcCys ligand, similarly to OA-coated NPLs. Electron diffraction confirmed the preservation of the crystal structure of zinc blende. EDX mapping confirmed homogeneity of L-AcCys ligand covering of the basal planes of CdSe NPLs (Figure 1e). In the case of ZnSe NPLs, ligand exchange preserved the flat triangles shape of sheets, similarly to the initial OLA- and OTA-coated NPLs.

### 3.2. FTIR Analysis of Ligand Coordination

**Figure 2:**
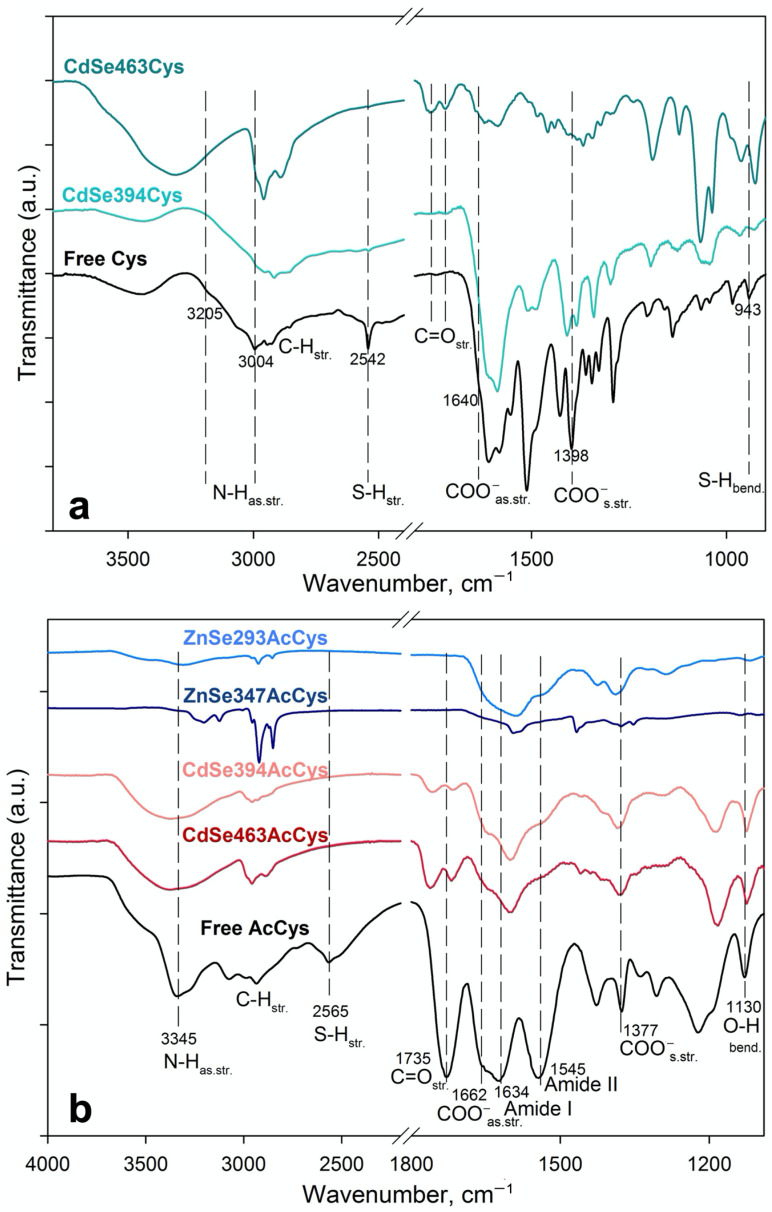
(**a**) FTIR spectra of L-cysteine capped 2.5 ML thick CdSe394Cys (dark green line) and 3.5 ML thick CdSe463Cys (turquoise line) samples. Black line shows the FTIR spectrum of pristine free L-cysteine reference. (**b**) FTIR spectra of N-acetyl-L-cysteine ligand capped 2.5 ML thick ZnSe293AcCys (blue line), 4 ML thick ZnSe347AcCys (dark blue line), 2.5 ML thick CdSe394AcCys (pink line), and 3.5 ML thick CdSe463AcCys (red line) samples. Black line shows the FTIR spectrum of pristine free N-acetyl-L-cysteine reference. Positions of the main vibration bans are marked by dashed lines. The spectra are offset for clarity.

Coordination of ligands on the basal planes of the NPLs was analyzed in detail by the FTIR method. To analyze the coordination of the L-cysteine ligand, samples of 2.5 and 3.5 ML thick CdSe NPLs were compared. Figure 2a shows the FTIR spectra of pure L-cysteine (black solid line) and cadmium selenide samples of different thicknesses coated with L-cysteine (2.5 ML—turquoise and 3.5 ML—green solid lines, respectively). In the L-cysteine spectrum, the bands observed at 2542 and 943 cm^−1^ are attributed to stretching and bending vibrations of S-H, respectively [25,26,27,28]. The bands at about 3205 and 3004 cm^−1^ correspond to N-H asymmetric stretching vibrations of the NH_3_^+^ group, whereas a group of bands in the range of 1550–1610 cm^−1^ corresponds to its bending vibrations [26,27,29,30]. The band at 1640 cm^−1^ (as a shoulder on the left side of the intense N-H_bend._ band) is attributed to COO^−^ asymmetric stretching, while the band at 1398 cm^−1^ is attributed to COO^−^ symmetrical stretching vibrations [27].

The bands at 2542 and 943 cm^−1^ of pure L-cysteine, assigned to S-H stretching and bending, are completely absent in the corresponding FTIR spectra of both cysteine-exchanged 2.5- and 3.5-ML samples, confirming the involvement of the sulfhydryl group in the binding to the NPLs basal planes. The bands at 1398 and 1612 cm^−1^ are ascribed to COO^−^ symmetrical and asymmetric stretching, respectively, and are observed only for a 2.5 ML thick sample, while for a thicker 3.5 ML sample, these vibrations almost completely disappear, but bands appear in the range 1700–1800 cm^−1^ and are attributed to the strong C=O stretching peak [18,25]. Thus, for a thin 2.5 ML sample, an (O-C-O)^−^ structure for the carboxylate group of ligand-cysteine is realized, where an electron is delocalized equally over the two oxygen atoms, and the carboxyl group does not participate in attachment to the surface; whereas, for a thick sample, the carboxylate group bounded to the surface cadmium cation forms a (O=C-O^−^) structure. The N-H bending (1550–1610 cm^−1^) and stretching (3205 and 3004 cm^−1^) bands are preserved for a 2.5 ML thick sample, but their intensities are largely weakened for a CdSe 3.5 ML thick sample. At the same time, a wide band at 3315 cm^−1^ appears for the latter, which can be attributed to NH_2_ stretching as confirmation of amines binding to a metal ion or to the surface of metal chalcogenide NPLs [18,25,31].

Thus, according to the FTIR data, for a thinner CdSe 2.5 ML sample coated with L-cysteine, a predominantly monodentate ligand coordination involving the sulfhydryl group is observed, while for a thicker one (CdSe 3.5 ML), another cysteine coordination involving all of the functional groups of the cysteine molecule is realized.

In the case of the N-acetyl-L-cysteine ligand, samples of 2.5 and 3.5 ML thick CdSe and 2.5 and 4 ML thick ZnSe NPLs were analyzed. The FTIR spectrum of free N-acetyl-L-cysteine (Figure 2b) gives bands at 3345 and 2565 cm^−1^, and a group of bands at 2900–3000 cm^−1^, which are attributed to the N–H, S–H and C-H stretching vibrations, respectively [32]. The bands at 1735, 1630, and 1545 cm^−1^ correspond to C=O stretching vibrations of the carboxylic group, Amide I and Amide II bands of the peptide group, respectively [32,33]. The band at 1130 cm^−1^ is ascribed to the bending mode of the −OH group of the carboxylic group [33]. The broad band around 3300–3600 cm^−1^ in the spectra may arise from the hydroxyl groups of H_2_O due to the absorption of water traces from the atmosphere.

The corresponding FTIR spectra of ZnSe and CdSe samples coated with N-acetyl-L-cysteine shows the disappearance of the characteristic bands of the S-H group, which indicates the successful bonding between the sulfur atoms and the metal atoms on the basal planes of NPLs.

The band at 1735 cm^−1^ is ascribed to C=O stretching of the carboxylic group and transforms for both samples of CdSe, indicating the binding of the COO^-^ group to the basal planes. The shift of the C=O band provides evidence of electrostatic interactions between carboxyl groups of N-acetyl-L-cysteine and the Cd atoms on the surface [34]. In the spectrum of ZnSe, no such band was observed; however, the bands of asymmetric and symmetric stretching vibrations of COO^-^ at 1650 and 1403 cm^−1^, respectively, become more pronounced [35,36]. At the same time, the vibration band of the bending mode of the -OH group (of carboxylic group) disappears. Thus, according to FTIR data, the carboxylate group is not involved in binding to the surface of ZnSe NPLs, but is present in the system as a charged (O-C-O)^−^ structure.

The bands of both N-H vibrations and amide vibrations of the N-acetyl-L-cysteine peptide group widen and lose their intensity for all samples, which indicates the binding of the amino group [35], similarly to the process already described above for this group of bands for the cysteine ligand.

Thus, according to the FTIR data, for CdSe samples coated with the N-acetyl-L-cysteine, all of the functional groups of the AcCys molecule are coordinated to the surface of NPLs, realizing various types of configurations, from mono- to probably tridentate. For ZnSe samples, binding is realized via sulfhydryl and amino groups, realizing mono- or bidentate configurations.

Note, in all the samples with thiolate ligands studied here, S-S vibration, usually appearing at approximately 573 cm^−1^ [28], was not observed, which indicates the absence of S-S binding between the ligands.

### 3.3. Analysis of Optical and Chiroptical Properties

**Figure 3:**
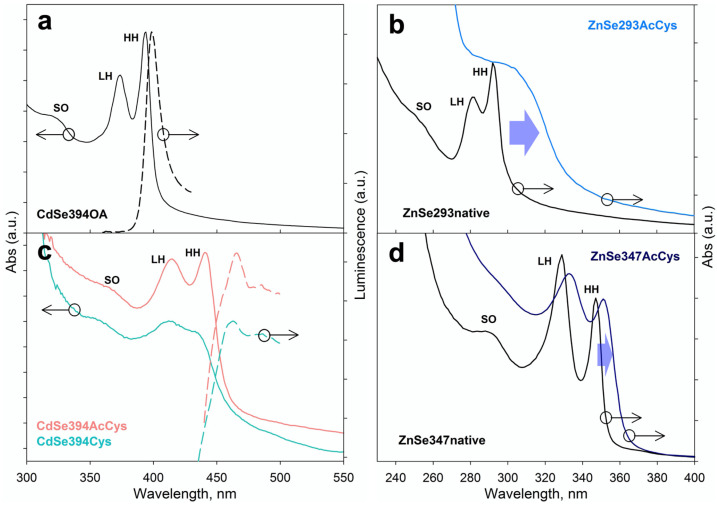
(**a**) Typical absorbance and luminescence spectra of CdSe394OA NPLs (black solid and dashed lines, respectively). (**b**) Absorbance spectra of ZnSe293OA NPLs (black dashed lines) and their modification after ligand exchange with AcCys ligands (ZnSe293AcCys, cyan line). (**c**) Modification of absorbance and luminescence spectra of CdSe394OA NPLs after ligand exchange with AcCys (CdSe394AcCys, pink lines) and Cys (CdSe394Cys, turquoise lines) ligands. (**d**) Absorbance spectra of ZnSe347OA NPLs (black dashed lines) and their modification after ligand exchange with AcCys ligands (ZnSe347AcCys, blue line). Blue arrows illustrate spectral shifts after ligand exchange.

Excitonic transitions of CdSe and ZnSe NPLs were analyzed by absorbance and luminescence spectroscopy depending on the type of ligands. Narrow exciton transitions at 394, 375, and 320 nm involving the heavy hole HH, light hole LH, and spin-orbit hole SO, respectively, were observed in the absorption spectrum of the as-synthesized CdSe394OA NPLs (Figure 3a), which agrees with the literature data [17,37,38]. The PL spectrum (dashed lines) shows a pronounced exciton luminescence band at 398 nm. Similar HH, LH, SO excitonic transitions are shown for thicker 3.5 ML CdSe463OA on Figure S4. In the case of as-grown ZnSe NPLs with OA ligand, narrow exciton HH, LH and SO transitions were observed (Figure 3b,d), similar to CdSe NPLs, but spectrally shifted to the UV region. Thin 2.5 ML ZnSe NPLs had a stronger shift at shorter wavelengths compared with the thicker 4 ML ZnSe NPLs, due to increase in the exciton confinement with decrease in thickness.

After ligand exchange of the CdSe394AcCys sample with L-AcCys ligand (Figure 3c), all excitonic maxima spectrally shifted about 40 nm to a longer wavelength. The observed decrease in the energy of exciton transitions should be attributed to the addition of the thiolate group of the L-AcCys ligand to the cadmium-rich basal planes, which agrees with the FTIR data. Binding of cadmium–sulfur leads to hybridization and an effective increase in the thickness of the nanoplatelets, which reduces the exciton confinement. A similar situation is observed upon addition of the L-Cys ligand, which also shifts exciton transitions to the longer wavelength spectral region. The almost identical spectral shift for the L-AcCys and L-Cys ligands confirms the involvement of the sulfhydryl group present in both ligands in binding to the NPL basal planes. It should be noted that luminescence is retained upon addition of ligands, although with a significant increase in the Stokes shift of about 40 nm compared to about 5 nm Stokes shift for the initial OA ligand (Figure 3a,c, dashed lines). A similar situation involving the retaining of clear HH, LH, SO excitonic transitions and their shift of wavelength after ligand exchange for thicker 3.5 ML CdSe463AcCys was observed (Figure S4). Note, for both 2.5 ML and 3.5-ML CdSe with thiolate ligands, a certain broadening of the exciton bands in the absorption spectra was observed, which correlates with the data of [17]. For ZnSe NPLs after ligand exchange with L-Cys ligand (Figure 3b,d), all excitonic maxima spectrally shift to a longer wavelength due to hybridization of ligand with NPL basal planes, similarly to CdSe NPL behavior. The strong broadening of exciton bands was found for both ZnSe293AcCys and ZnSe347AcCys samples, compared to the nanoplatelets with OA ligands, which should indicate a stronger exciton-phonon coupling in the case of sulfur binding, as was previously observed for cadmium chalcogenides [21,22].

**Figure 4:**
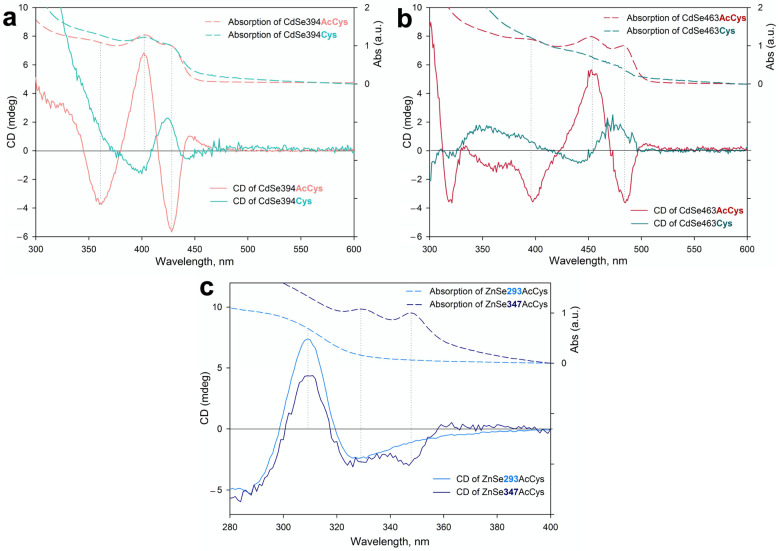
(**a**) CD spectra of 2.5 ML thick CdSe394AcCys and CdSe394Cys samples modified by N-acetyl-L-cysteine (pink line; L-AcCys) and L-cysteine (turquoise line; L-Cys), respectively. Absorbance spectra are shown by dashed lines for comparison. (**b**) CD spectra of 3.5 ML thick CdSe463AcCys and CdSe463Cys samples modified by N-acetyl-L-cysteine (red line; L-AcCys) and L-cysteine (green line; L-Cys), respectively. Absorbance spectra are shown by dashed lines for comparison. (**c**) CD spectra of 2.5 ML thick ZnSe293AcCys (blue line) and 4 ML thick ZnSe347AcCys (dark blue line) samples modified by N-acetyl-L-cysteine. Absorbance spectra are shown by dashed lines for comparison. Pointed lines show correspondence between CD and absorbance bands.

The chiroptical properties of NPL samples coated with chiral ligands was studied by CD spectroscopy and presented on Figure 4. A typical CD spectrum of an N-acetyl-L-cysteine-coated CdSe394AcCys sample (Figure 4a) shows five distinct, sign-alternating bands in the spectral range of 300–500 nm, supporting the influence of the chiral ligand on the induced preference in absorbance of right- or left-hand circularly polarized light by an NPL stereoisomer. The positions of the CD bands correlate well with the LH, HH, and SO transitions in the absorption spectra, confirming the exciton nature of the induced CD bands. Replacing the chiral ligand on L-cysteine changes the spectrum. The CD spectrum of an L-cysteine-coated CdSe394Cys sample also shows sign-alternating bands in the spectral range of 300–500 nm; however, they are inverted, mirror-like, to the spectrum of N-acetyl-L-cysteine-capped samples. It should be noted that the standard situation is when the CD signal is inverted upon transition from the L- to D-stereoisomer of ligands. In our case, inversion occurs for ligands of the same absolute L-configuration. The change in coordination of the chiral ligand on the surface seems to be responsible for the CD signal inversion, as a result of the change in the orientation of coupled dipole transition moments of the chromophore group of the ligand and the exciton transition of the nanoplatelets. As was shown in [19] for L/D-cysteine-capped 3.5 ML CdSe nanoplatelets, variation of crystal structures from cubic to hexagonal inverts the CD signal. The same effect of the ligand coordination was proposed in [18] for water-dispersed L/D-cysteine-capped CdSe quantum dots. Also, the authors of [39] reported CD spectra inversion upon change from L-cysteine to N-acetyl-cysteine for water-dispersed CdSe and CdS quantum dots. The authors attributed the effect to the different binding arrangements of the chiral ligands. In the case of our 2.5 ML CdSe NPLs with cubic crystal structure, CD signal inversion is clearly supported by the change in the coordination of the chiral ligands: the monodentate coordination of cysteine ligands changed to the coordination of all of the functional groups of the acetylcysteine, bonded to the same zinc blend basal planes, as shown by FTIR data. More sterically bulky compared to Cys ligand, AcCys ligand probably coordinates by the sulfhydryl and the carboxylic groups binding to different Cd surface atoms replacing carboxylic groups of native OA ligands, while Cys ligand coordinates via only the sulfhydryl group. This situation is opposite to the scenario of AcCys and Cys ligand coordination to the surface of spherical quantum dots [39] with bidentate Cys ligand and monodentate AcCys ligand coordination. Indeed, in the CD spectra, we observe sign-alternating bands opposite to the data of [39], confirming the inversion in ligand binding. This result should be attributed to dense packing of ligands on NPL basal planes [17] compared to more sterically free ligands on the surface of quantum dots. At the same time, a stronger CD signal was observed for the N-acetyl-L-cysteine-coated NPL sample.

The same behavior was observed for thicker 3.5 CdSe NPLs (Figure 4b). A shift of the CD bands to shorter wavelength in the CD spectra was found simultaneously with a shift of the exciton bands in the absorbance spectra. The positions of sign-alternating CD bands correlate with the LH, HH, and SO exciton transitions, which confirms the induction of chirality in the exciton system. At the same time, an increase in NPL thickness reduces the intensity of the CD signal compared to a 2.5 ML sample, indicating the decrease of coupling of the exciton dipole and the dipole of the chromophore group of the ligand, and the strength of quantum confinement in the thicker sample. However, replacing the N-acetyl-L-cysteine ligand to L-cysteine also inverted the CD spectrum in the same way as for the 2.5 ML sample. That also be attributed to change in the ligand coordination shown by FTIR spectroscopy.

Some different behavior was observed for wurtzite ZnSe NPLs modified by N-acetyl-L-cysteine (Figure 4c). CD spectra demonstrate less pronounced CD bands without a clear alternation of signs. Nevertheless, a certain correspondence can be distinguished between the positions of the CD bands and the absorption bands, which confirms the induction of chirality. This behavior may be the result of a stronger broadening of the exciton bands and, at the same time, less efficient binding of thiolate ligands to the basal planes of nanoplates due to the lower efficiency of zinc-sulfur binding compared with cadmium-sulfur binding. Simultaneously, the CD signal for thinner 2.5 ML NPL has a higher intensity compared to that of 4 ML NPL, which is the same effect of NPL thickness as observed for CdSe NPLs.

To analyze the rotary strength of chiral CD transitions, we calculated the dissymmetry g-factor. Table 1 presents the observed spectral positions of the CD bands and the calculated values of the dissymmetry factor for each CD band for ZnSe and CdSe samples. As can be seen, the dissymmetry factor for the acetylcysteine ligand exceeds the value for cysteine, which can be attributed to more efficient binding and favorable coordination involving all three functional groups of the molecule compared to the monodentate coordination of cysteine. Also, for the thinnest of both CdSe and ZnSe NPLs, the maximum dissymmetry factor of up to (2–3) × 10^−3^ was found, due to the maximum hybridization of the dissymmetric center of the ligand and the exciton system of the nanoplatelets for the minimum thickness [17,40], which exceeds most earlier reported values. Thus, these approaches seem to be useful to the development of a new chiral colloidal 2D semiconductor material.

## 4. Conclusions

This work demonstrates that the exchange with cysteine-based ligands on the basal planes of atomically thin CdSe and ZnSe NPLs leads to an effective induction of chirality with distinct bands of CD exciton transitions. We successfully grew atomically thin 2.5 and 3.5 ML (0.6–0.9 nm thick) CdSe with zinc blende modification and 2.5 and 4 ML (0.6–1.2 nm thick) ZnSe nanoplatelets with wurtzite modification covered by long-chain ligand by the colloidal method in ODE–OA–cadmium acetate and ODE–OLA–OTA–zinc oleate systems, respectively. The developed protocols for the exchange of native long-chain ligands for cysteine-based ligands in organic solvents allowed complete capping of NPL basal planes with chiral ligands, while maintaining the morphology and crystal structure. Careful analysis of ligand coordination showed that L-cysteine is coordinated monodentate, while for N-acetyl-L-cysteine, all three functional groups are involved in coordination. Exchange with chiral ligands induces a shift of excitonic bands due to the thiolate group hybridization, together with the appearance of strong CD bands in CD spectra. The values of the dissymmetry factor for ultimate atomically thin nanoplatelets exceed those for thicker samples. At the same time, the dissymmetry factor for the N-acetyl-L-cysteine ligand exceeds the value for L-cysteine, confirming that the N-acetyl-L-cysteine is of interest for inducing chirality, along with the commonly used L-cysteine. Exchange of the N-acetyl-L-cysteine ligand to L-cysteine leads to an inversion of the CD spectra, which emphasizes the reorientation of the molecular dipoles of the chromophore of the ligand and the exciton transition in the core of the nanoplatelet. Our work gives new strategies for the synthesis of chiral 2D nanostructures. We believe that, due to a thickness of only a few atoms and 2D electronic structure, chiral atomically thin CdSe and ZnSe NPLs could offer great opportunities for the development of chiroptical sensors, enantioselective separation, display technologies, and other potential applications.

## Figures and Tables

**Table 1:** Spectral position λ_CD_ and dissymmetry g-factor for the main circular dichroism bands of CdSe and ZnSe samples with different thickness, covered with L-Cys or L-AcCys ligands.

Sample	Ligand	𝜆_CD_, nm	g-Factor (×10^−3^)
CdSe394	*AcCys*	403	1.30
		428	−1.85
	*Cys*	402	−0.37
		425	0.52
CdSe463	*AcCys*	453	1.13
		483	−1.2
	*Cys*	443	−0.13
		473	0.35
ZnSe293	*AcCys*	309	3.22
ZnSe347	*AcCys*	330	−0.77
		348	−0.82

## Data Availability

The data that support the findings of this study are available from the corresponding author upon reasonable request.

## Supplementary Materials

The following supporting information can be downloaded at: https://www.mdpi.com/article/10.3390/ma16031073/s1, Figure S1: TEM images of as-synthesized CdSe463OA and after ligand exchange with L-AcCys, Figure S2: TEM images of as-synthesized ZnSe347OA and stack of triangle ZnSe293OA NPLs covered with oleic acid ligands, Figure S3: FTIR spectra of free N-acetyl-L-cysteine, 2.5 ML thick CdSe394AcCys and CdSe394OA samples and FTIR spectra of 2.5 ML thick ZnSe293OA, ZnSe293AcCys samples and free N-acetyl-L-cysteine, Figure S4: Typical absorbance spectra of CdSe463OA NPLs and its modification after ligand exchange with AcCys; luminescence spectra of CdSe NPLs coated with AcCys and Cys.
