# Supplementary material for: Induction of Chirality in Atomically Thin ZnSe and CdSe Nanoplatelets: Strengthening of Circular Dichroism via Different Coordination of Cysteine-Based Ligands on an Ultimate Thin Semiconductor Core
Source: Materials (Basel). 2023 Jan 26;16(3):1073. doi: 10.3390/ma16031073 (PMC9920291; doi:10.3390/ma16031073)
Supplement: Supplementary file 1 [file materials-16-01073-s001.zip › materials-2124335-supplementary.pdf]

Supplementary Materials

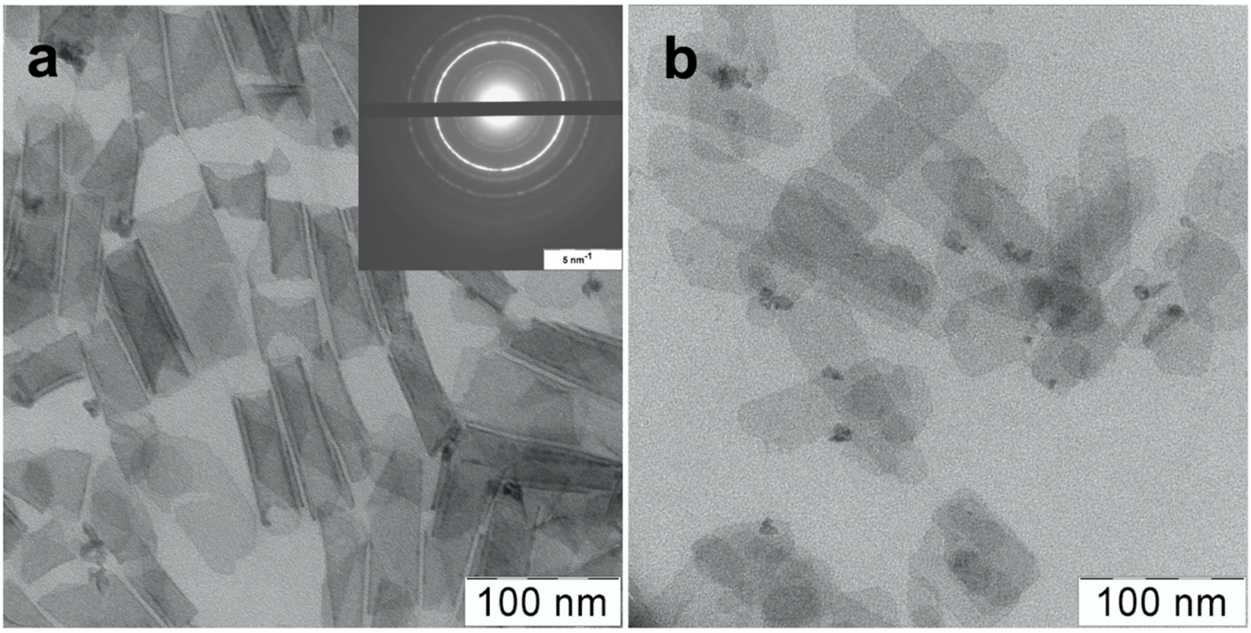

Figure S1. TEM images of (**a**) as-synthesized scroll-like CdSe463OA and (**b**) the same NPLs after ligand exchange with L-AcCys. Insert shows electron diffraction image.

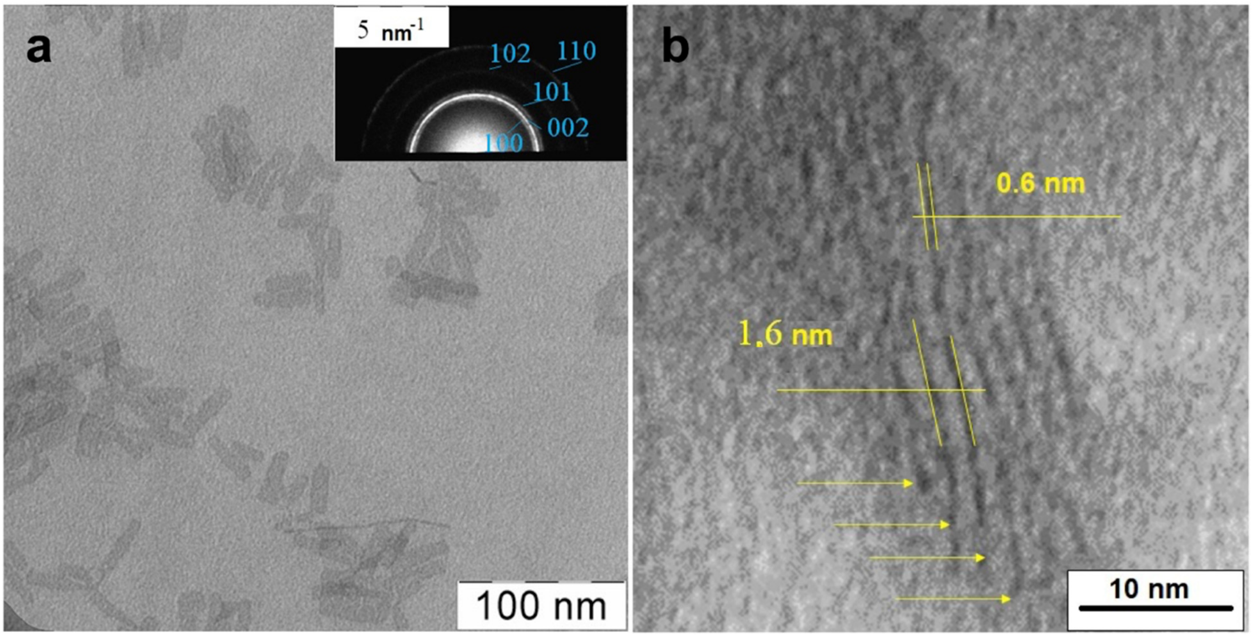

Figure S2. TEM images of (**a**) as-synthesized rectangular ZnSe347OA (insert shows electron diffraction image); (**b**) stack of triangle ZnSe293OA NPLs covered with oleic acid ligands.

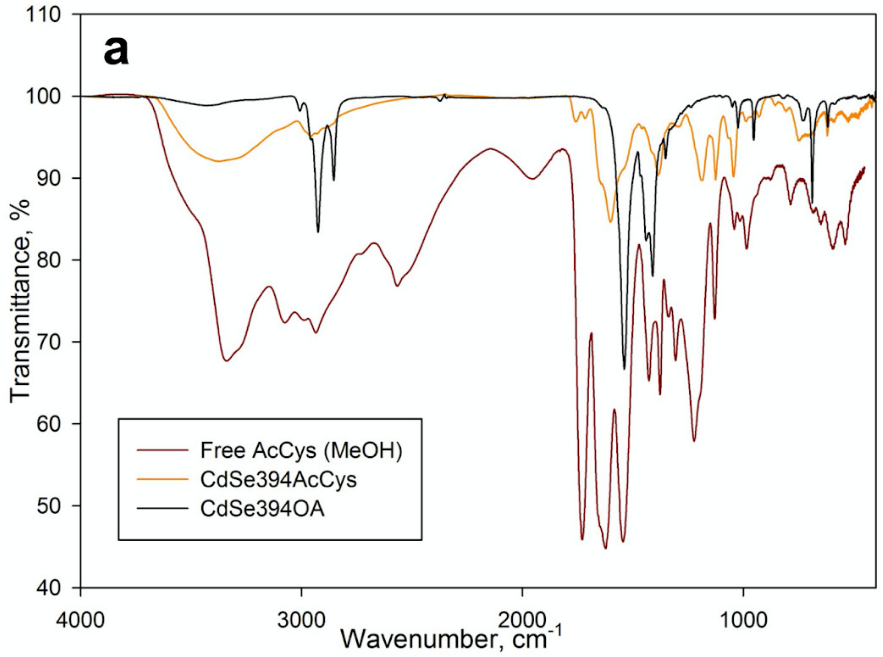

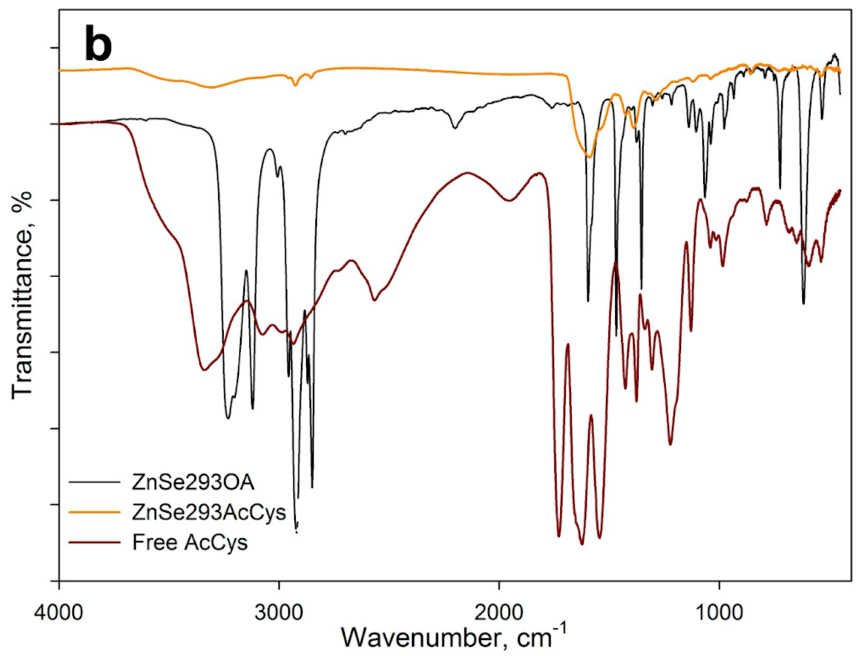

Figure S3. (**a**) FTIR spectra of free N-acetyl-L-cysteine (red line), 2.5 ML thick CdSe394AcCys (orange line) and CdSe394OA (black line) samples; (**b**) FTIR spectra of 2.5 ML thick ZnSe293OA (black line) and ZnSe293AcCys (orange line) samples, red line is for free N-acetyl-L-cysteine.

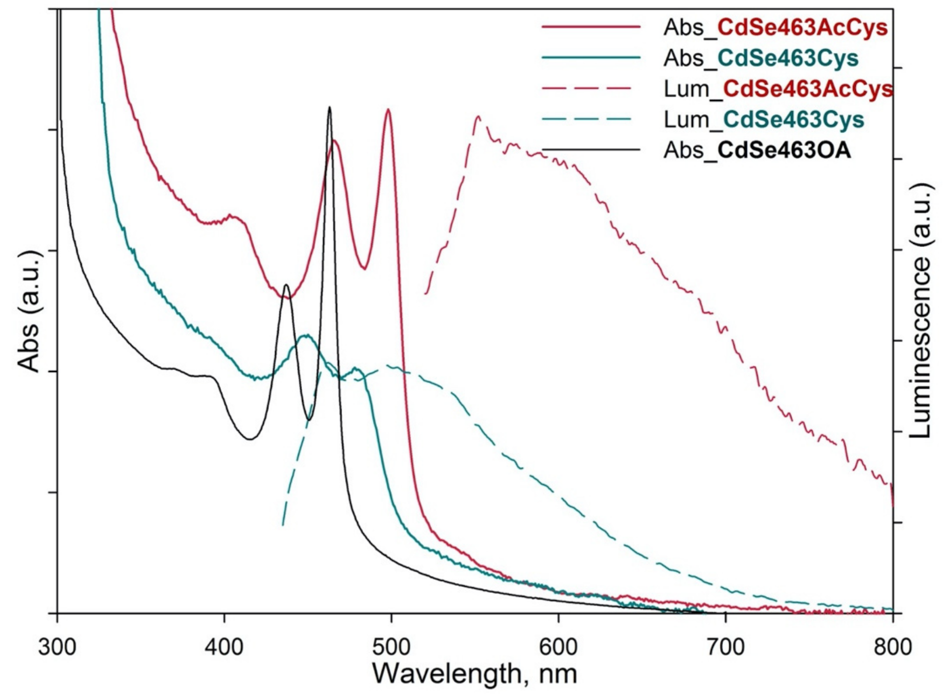

Figure S4. Typical absorbance spectra of CdSe463OA NPLs (black solid line) and its modification after ligand exchange with AcCys (CdSe463AcCys, red solid line) and Cys (CdSe463Cys, green solid line) ligands; luminescence spectra of CdSe NPLs coated with AcCys and Cys are represented as dashed lines (red and green, respectively).
